# 1760. Selection of appropriate inpatients for outpatient parenteral antimicrobial therapy (OPAT) during the COVID pandemic

**DOI:** 10.1093/ofid/ofac492.1390

**Published:** 2022-12-15

**Authors:** Angela Y McKnight, Angelike P Liappis, Ayne Adenew

**Affiliations:** DC VA Medical Center, Washington, District of Columbia; DCVA Medical center/The George Washington University, Washington, District of Columbia; DCVA Medical Center, washington, District of Columbia

## Abstract

**Background:**

Providing a safe discharge in patients appropriate for outpatient parenteral antimicrobial therapy (OPAT) became an important focus of the inpatient to outpatient transition during the pandemic. Hospital beds were needed for ill patients with COVID; transfers in and out of facilities and interfacility infection became issues for long-term care (LTCF) and short-term nursing facilities (SNFs) in the US during the pandemic. We examine the traditional barriers, including age, and outcomes of patients selected for OPAT initiation during the pandemic period.

**Methods:**

Washington DC VAMC (WDVAMC) is an urban medical center with 220 acute and LTC beds. At the start of the pandemic, a multidisciplinary OPAT program was initiated and led by ASP Nurse Practitioner (NP), supported by ASP Pharmacist and ASP/ID Physician. OPAT included care continued at home, SNF, LTC and hemodialysis locations; the OPAT program focus was on appropriate selection, post-discharge safety monitoring, outpatient follow-up at completion, tracking and outcome determination (30D, 90D readmissions). OPAT related readmissions related to drug or opat-indicated infections.

**Results:**

Between April 2020-2022, 157 unique OPAT discharges (10.4 OPAT/1,000 admissions 2020 and 4.8 OPAT/1,000 admissions 2021); the majority were offered OPAT at home (47%). Mean duration OPAT 22±13days over that period; most frequent indication osteomyelitis and BSI. OPAT related readmissions were infrequent at 30D (7%) and 90D (1%). The mean age was 67±13y with 5% >90yrs, 21% >75yrs and 58% >65yrs. In the pandemic 92% completed OPAT; compared to younger group those >75y, rates of completion (93% vs 92%, P=NS) and mean duration of OPAT were similar however a modest increase in 90D OPAT-related readmission (1.2% vs 0%, P=0.04). The only two deaths prior to completion of OPAT occurred in those >75y.

**Conclusion:**

Transitions to OPAT are required for serious infections in hospitalized patients of all ages, however transitions to SNF and LTC were limited at times during the pandemic. We found OPAT can be delivered safely, regardless of age during a pandemic state with careful coordination between stakeholders and a systematic process to monitor outcomes and track patients, particularly for the elderly.

**Disclosures:**

**All Authors**: No reported disclosures.

